# Metabolomic Analysis of the Urine from Rats with Collagen-Induced Arthritis with the Effective Part of *Caulophyllum robustum* Maxim

**DOI:** 10.1155/2021/5580341

**Published:** 2021-05-26

**Authors:** Shaowa Lü, Mingtao Zhu, Qiaoxin Guo, Dan Xu, Yuyan Guo, Guoyu Li, Qiuhong Wang, Haixue Kuang

**Affiliations:** ^1^Key Laboratory of Chinese Materia Medica (Ministry of Education), Heilongjiang University of Chinese Medicine, Harbin 150040, China; ^2^Pharmaceutical College, Harbin University of Commerce, Harbin 150086, China; ^3^Science of Processing Chinese Materia Medica, School of Traditional Chinese Medicine, Guangdong Pharmaceutical University, Guangzhou 510024, China; ^4^Heilongjiang University of Chinese Medicine, Harbin 150040, China

## Abstract

Rheumatoid arthritis (RA) is a chronic autoimmune disease with high incidence and high disability and recurrence rates. *Caulophyllum robustum* Maxim (*C. robustum*) is a traditional Chinese medicine (TCM) with main effective parts (CRME) commonly used for RA treatment. To explore the mechanism of CRME in RA, we used metabolomics to investigate the effect of CRME intervention on urine metabolism in rats with collagen-induced arthritis (CIA). CIA rats were randomly divided into normal control, CIA model, and CRME groups. A metabolomics approach, using Ultra-Performance Liquid Chromatography-Quadrupole-Time-of-Flight/Mass Spectrometry, was developed to perform urinary metabolic profiling. Differential metabolites were identified by comparing the CIA model and CRME groups. Preliminarily, 56 significant differential metabolites were identified in urine, and 20 metabolic pathways were disturbed by the CIA. The amount of 16 different metabolites changed in urine after CRME intervention. The production of these metabolites involves tryptophan, tyrosine, energy, cholesterol, and vitamin metabolism. CRME has anti-inflammatory and immunosuppressive effects in CIA model rats. By examining the endogenous metabolite levels, we identified potential CRME targets and pathways involved in the treatment of RA. The results of our metabolic studies indicate that CRME regulates amino acid, vitamin, energy, and lipid metabolism pathways to treat RA and may provide a new explanation for the anti-RA mechanism of CRME.

## 1. Introduction

Rheumatoid arthritis (RA) is a chronic autoimmune and systemic inflammatory disease that results in joint injury, disability, and decreased quality of life and has distinct signatures of persistent synovitis and hyperplasia [1]. Worldwide, approximately 0.24% of the population suffers from RA. Although RA has been studied for many years, the pathogenesis of this disease is still incompletely understood. Currently, there are no ideal methods or medicines to cure RA. However, it is known that endogenous metabolism is changed in RA patients [2]. Moreover, studies and clinical test results indicate that traditional Chinese medicine (TCM) can play important roles in alleviating the symptoms of RA [3].

*Caulophyllum robustum* Maxim (*C. robustum*) is also known as “Hong Mao Qi”. *C. robustum* is widely distributed in northeast, northwest, and southwest China, and the main effective parts of this plant are its roots and rhizomes. *C. robustum* can dispel wind, remove obstruction in the meridians, promote blood circulation, and stop the pain, and it has been used to treat rheumatic arthralgia, irregular menstruation, stomachache, and abdominal pain [4–6]. *C. robustum* Maxim extract (CRME) mainly contains saponins and alkaloids [7], and spectrum-activity relationship results indicate that multiple CRME components have anti-inflammatory and immunosuppressive effects [8]. CRME has obvious dose-dependent therapeutic effects in RA animal models where it can regulate the levels of tumor necrosis factor and interleukin family inflammatory cytokines [9] and regulate cellular and humoral immunity [10, 11]. CRME also can protect cardiocytes [12], slow the formation and proliferation of synovial pannus [13], and reduce articular cartilage and bone erosion and destruction [13]. Together, these results indicate that CRME is effective in the prevention and treatment of RA and immune hyperactivity. However, the mechanism of TCM is complex, and it is difficult to elucidate the effects of TCM at the molecular level.

Metabonomics is a new omics technology that combines systems biology, genomics, and proteomics [14]. Metabolomics is mainly used to determine endogenous metabolites in biological samples using qualitative and quantitative modern analytical techniques (including LC-MS, GC-MS, and NMR) [15]. Changes in the endogenous metabolites produced before and after TCM administration were investigated using chemical informatics (PCA, PLS-DA, and OPLS-DA) [14, 16, 17]. Using this approach, differentially produced metabolites can be used as biomarkers of the pathologic and physiologic body state, can illustrate the efficacy of TCM pharmacodynamic material, and uncover the mechanism of TCM action [18].

We used a combination of metabonomics and modern analytical techniques to explore the effects of CRME on the urine metabolic profiles in collagen-induced arthritis (CIA) rats. Then, we explored the role of CRME in regulating small molecular metabolites in the body and metabolic pathways by examining the changes in biomarkers. Research of the related metabolic pathways was used to explore the mechanism of CRME action, its anti-inflammatory effects, and its targets in the body. This molecular-level understanding can guide the clinical application of CRME.

## 2. Materials and Methods

### 2.1. Chemicals and Reagents

CRME was purchased from Suiling County of Heilongjiang and was identified by Prof. Lü Shaowa (Heilongjiang University of Chinese Medicine, China). Bovine type II collagen (CII, Lot DA0807LA14) was purchased from Shanghai Yuanye Biotechnology Co. Ltd (Shanghai, China). Complete Freund's adjuvant (CFA, Lot SLBJ2845 V) and Incomplete Freund's adjuvant (IFA, Lot SLBL0210 V) were purchased from Sigma Co., (USA). Glacial acetic acid was purchased from Tianjin Fuyu Fine Chemical Industry Co., Ltd. (Tianjin, China). Tripterygium was purchased from Hunan Qianjin Pharmaceutical Co. (Hunan, China). Distilled water was purchased from Guangzhou Watson's Food and Beverage Co. Ltd. (Guangzhou, China). Formic acid was purchased from Shanghai Anpel Experimental Technology Shanghai Co., Ltd. (Shanghai, China). Acetonitrile was purchased from Thermo Fisher Scientific Co., Ltd. (Shanghai, China).

### 2.2. Animals

A total of 24 specific Wistar female rats, weighing 190 ± 10 g, were obtained from the Comparative Medicine Centre of Heilongjiang University of Chinese Medicine (Heilongjiang, China) with permission number SCXK (HEI) 2013–004. All animals were housed in controlled laboratory conditions at 25°C with a 12 h light/dark cycle and 60% humidity. Rats had free access to water and standard pellet feed and were acclimatized in metabolic cages for 7 days prior to experimentation. Animal experiments were approved by the laboratory animal ethics committee of Heilongjiang University of Chinese Medicine (Grant No. 2017032401).

### 2.3. CRME Preparation

*C. robustum* was purchased from the Heilongjiang Province of China and certified by Professor Shaowa Lv (College of Pharmacy, Heilongjiang University of Traditional Chinese Medicine). A voucher specimen (20150301) was stored in the College of Pharmacy, Heilongjiang University of Traditional Chinese Medicine. The original medicinal materials for CRM (100 g) were extracted twice with 70% ethanol (v/v) at reflux, and the extract was placed in an AB-8 macroporous adsorption resin column and collected. The 70% eluate obtained was dried by evaporation in a rotary evaporator, and the solvent was recovered to obtain the effective part of *C. robustum* Maxim (CRME). The six main CRME components, CaulosideH, LeonticinD, CaulosideG, CaulosideD, CaulosideB, and CaulosideC, were determined by high-performance liquid chromatography (HPLC). The results showed that the CRME was composed of 6.21%, 5.14%, 28.88%, 10.19%, 10.19%, and 2.26% for CaulosideH, LeonticinD, CaulosideG, CaulosideD, CaulosideB, and CaulosideC, respectively.

### 2.4. Model Construction and Drug Administration

Wistar rats were selected as subjects. The appropriate amount of CII was precision weighed, and a 2 mg/ml solution was generated by dissolving in glacial acetic acid. The solution was stored at 4°C overnight. The collagen solution was emulsified with CFA in a 1 : 1 ratio on the first day of immunization and fully emulsified in the ice bath environment, and eventually, the emulsion droplet dripped into the water is not dispersed as appropriate. Rats were intradermally injected with the CII emulsion (0.2 mL) at the base of the tail at three equidistant locations, and the first basic immunization was recorded as d0. Collagen solution was emulsified with IFA in a 1 : 1 ratio on the second day of booster immunization. A secondary immunization, or boost, using 0.2 mL of the CII emulsion was given in the same way on day 7. Rats in the normal group were not treated and had normal feeding.

Rats were divided into normal control group, CIA model group, and CRME group (*n* = 8 in each group). Gavage was started on the day of the second immunization. The doses were CRME 69.58 mg/kg for 30 consecutive days. The blank group and the CIA model group were given equal volumes of distilled water.

### 2.5. Handling of Animal Samples

After successful modeling of CIA rats, all experimental rats were continuously administered CRME for 30 days. Rats had free access to water, without a standard diet, for a 12 h period on days 10, 20, and 30 during which time they were placed into metabolic cages for urine sample collection. The fresh urine samples with sodium azide were stored at −80°C immediately before use. All urine samples were centrifuged at 5000 rpm for 10 min, and the supernatant was stored at −80°C until required for analysis. Prior to analysis, 150 *μ*L of urine from each sample was vortex-mixed with 150 *μ*L cold methanol acetonitrile (2 : 1, v/v) solution and 10 *μ*L internal standard (2-chloro-L-phenylalanine, 0.3 mg/mL, methanol configuration), subjected to ultrasonic extraction for 5 minutes and left to stand for 20 minutes at −20°C. Urine samples were collected after centrifugation at 14,000 rpm for 10 min at 4°C and weighed. Clear supernatant (200 *μ*L) was transferred to a sampling vial for UPLC- MS analysis.

### 2.6. UPLC-Q-TOF/MS Conditions

Chromatographic separation was performed on a Waters UPLC I-class system equipped with a binary solvent delivery manager and a sample manager, coupled with a Waters VION IMS Q-TOF Mass Spectrometer equipped with an electrospray interface (ESI; Waters Corporation, Milford, USA). The ACQUITY UPLC BEH C18 column (100 mm × 2.1 mm i.d., 1.7 *µ*m; Waters Corporation) was maintained at 40°C for urine analysis. The gradient conditions for urine samples were as follows: 0–1.0 min: 1%–1% B; 1.0–5.5 min: 1%–20% B; 5.5–6.0 min: 20%–30% B; 6.0–8.5 min: 30%–35% B; 8.5–10.5 min: 35%–70% B; 10.5–11.0 min: 70%–100% B; 11.0–13.0 min: 100%–100% B; 13.0–13.1 min: 100%–1% B; and 13.1–15.0 min: 1%–1% B. The flow rate was set at 0.40 mL/min, and the injection volume was set as 3 *μ*L for all analyses.

Mass spectrometric data were collected using a Mass Spectrometer equipped with an ESI source operating in positive or negative ion mode. The sample injection and the collision voltage were 1.0 kV and 40 V, respectively. The ionic source and dissolving temperatures were 120°C and 500°C, respectively, and the desolvation gas flow was 900 L/h. Centroid data was collected from 50 to 1,000 m/*z* with a scan time of 0.1 s and an interscan delay of 0.02 s over a 13 min analysis time.

### 2.7. Urine Metabolic Profiling Analysis

Baseline filter, peak identification, integration, retention time correction, peak alignment, and normalization were performed by the metabolomics processing software of the instrument Progenesis QI (Waters Corporation) using raw data to obtain a data matrix with retention time, mass-to-charge ratio, and peak intensity. The normalized urine sample data matrix was imported into the SIMCA-P + 14.0 software package (Umetrics, Umea, Sweden) for unsupervised principal components analysis (PCA) to observe the overall sample distribution and the stability of the analysis process. Then, the supervised (orthogonal) partial least square method, (O)PLS-DA, was used to distinguish overall metabolic profile differences and identify metabolite differences between groups. Variables with variable important in projection (VIP) scores greater than 1 were considered differential variables. To prevent the model overfitting, the quality of the model was investigated using seven interactive verification cycles and 200 response sequencing tests.

### 2.8. Differential Metabolite Screening and Identification

Screening the metabolite differences between groups (VIP > 1, *p* < 0.05, Fold Change (FC) > 1.2) was achieved using multidimensional analysis of PLS-DA and single-dimensional analysis (Student's *t*-test) methods. Differential metabolites were selected for qualitative analysis using http://www.hmdb.ca/and self-built databases.

**Figure 1:**
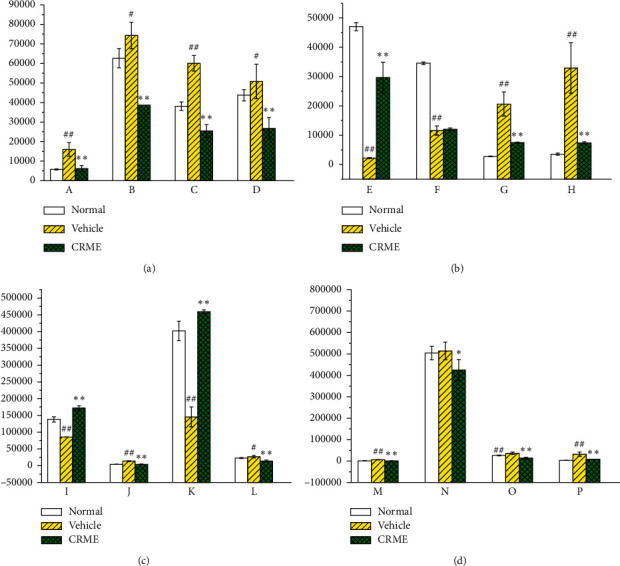
The relative content of 16 reversed metabolites in urine samples. ^#^*p* < 0.05, ^#^*p* < 0.01, compared with the blank group. ^*∗*^*p* < 0.05, ^*∗∗*^*p* < 0.01, compared with the model group. A: N1-Methyl-4-pyridone-3-carboxamide; B: 2,4-Diaminobutyricacid; C: 7-Hydroxy-3-oxocholanoic acid; D: alpha-CEHC; E: hydroquinone; F: 2-indolecarboxylic acid; G: 17-beta-estradiol-3-glucuronide; H: 5b-cholestanol; I: hippuric acid; J: D-ribose; K: benzoic acid; L: monoisobutyl phthalic acid; M: 5-b-cholestane-3a,7a,12a-triol; N: L-tyrosine; O: indoxyl; P: stigmastanol.

According to the variable projection importance (VIP) parameter in the PLS-DA model, the potential biomarkers were evaluated, and the different variables of VIP > 1 were selected according to the order of VIP, and then, the compounds with significant differences among the three groups were screened by *t*-test. Only the different variables with *p* < 0.05 were considered as potential biomarkers. According to the matching results and HMDB metabolite database search, as shown in Figure 1 and Table 1, 16 metabolites were identified, which were preliminarily considered as potential biomarkers induced by the effective part of *Caulophyllum robustum* Maxim.

### 2.9. Effect of CRME on Urine Metabolism Profiles and Differential Metabolites Identified in CIA Rats

The changes in differential metabolites in the last urine collection of experimental rats administered CRME were analyzed to analyze the substances with callback during the treatment for analyzing the effect of effective parts of CRME on the differential metabolites in the urine of rats. The metabolic pathways of differential metabolites with callbacks were analyzed and determined.

## 3. Results

### 3.1. Assessment of the CIA Model and the Anti-Inflammatory Effects of CRME

**Figure 2:**
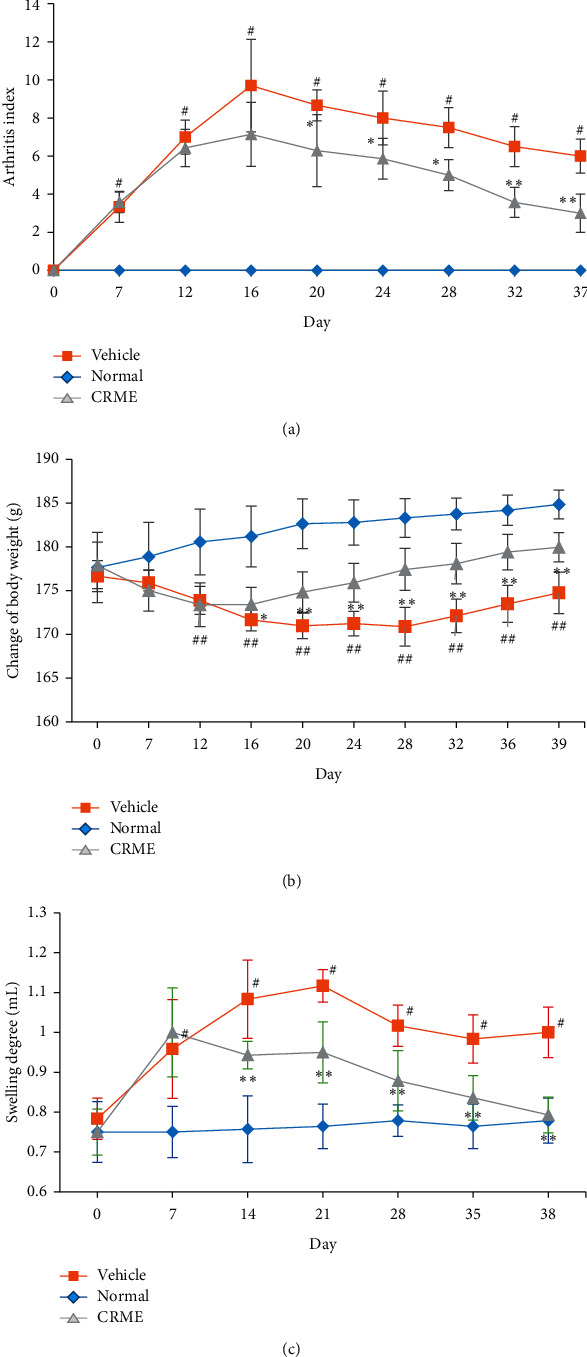
The medicinal effects of CRME TCA in CIA model rats (*x* ± *s*,*n* = 8). (a) Arthritis index score. (b) Weight changes. (c) Foot volume changes. ^#^*p* < 0.01, compared with the normal control group. ^*∗*^*p* < 0.05, ^*∗∗*^*p* < 0.01, compared with the model group.

The rats of the normal control group had normal feeding and behavior, and their weight gradually increased. Rats of the CIA group showed a trend of decreasing weight, and their activity decreased after 12 days (*p* < 0.01). Compared with the model group, the bodyweight of the rats in each group increased over the period of CRME administration (*p* < 0.05) (Figure 2(a)). The arthritis index (AI) in CIA rats that are administered CRME was examined. The AI value of rats in each group reached the maximum on day 16. Compared with the CIA model group, the AI values of rats in each group showed a decreasing trend from the 10^th^ day after administration, and the AI values at different time points in each administration group, including the CRME group (*p* < 0.05), significantly differed from those in the model group (*p* < 0.01) (Figure 2(b)). Compared with the blank group, the swelling of the right hind limb in the CIA model group was most evident on day 10 (*p* < 0.01). Compared with the model group, the foot volume of rats in each group began to significantly decrease after 7 days of CRME administration (*p* < 0.05 or *p* < 0.01) (Figure 2(c)).

### 3.2. Spectral Data Analysis

A visual inspection of the base peak ion flow chromatogram (BPI) was performed for all samples. This revealed that all samples had strong signal strength, large peak capacity, and good retention time. Urine samples from rats in the CRME group were analyzed after 30 days of CRME administration. BPI chromatograms in positive and negative ion modes are shown in Figure S1.

### 3.3. Metabolic Profiling Analysis of Urine

**Figure 3:**
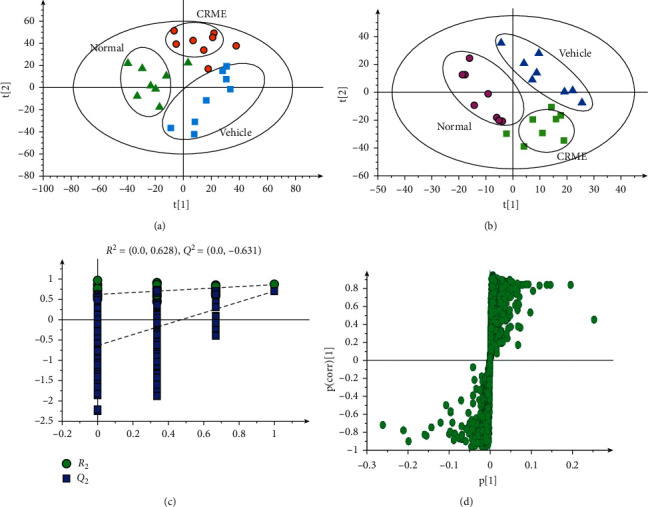
PCA (a), PLS-DA (b), and PLS-DA (c) model validation and S-plot score plots (d) based on the normal and CIA model group rat urine.

A mathematical model was established for urine samples in blank and model groups using PCA and PLS-DA. The score chart (Figure 3(a)) and PCA analysis parameter values (*R*^2^X = 0.655) reveal a total metabolic difference between blank and CIA model groups, and the variation of the sample within the group is greater. These results show that the analysis process was stable, and the physiological state of the model group obviously differed from that of the blank group. The PLS-DA score plots (*R*^2^X = 0.537, *R*^2^Y = 0.871, *Q*^2^ = 0.649) of urine samples indicated that the mathematical model is reliable and stable and can be used to find potential differential metabolites. Compared with the normal group, the samples from model rats metabolically differed based on the PLS-DA score plot (Figure 3(b)), indicating that the metabolism of endogenous substances differs in rats after injecting collagen and leads to significant changes in the metabolic fingerprint of urine samples in rats.

The closer the slope of the normal *R*^2^Y and *Q*^2^Y lines is to the horizontal line, the more likely the model is to be overfitted. Additionally, *Q*^2^ is generally required to be less than zero when using the RPT test (Figure 3(c)). In this experiment, *Q*^2^ = −0.631 of urine samples, indicating that the model is well-fitted. The points at both ends of the s-type are potential biomarkers and are determined by VIP values greater than 1.0 in the S-plot diagram (Figure 3(d)).

**Figure 4:**
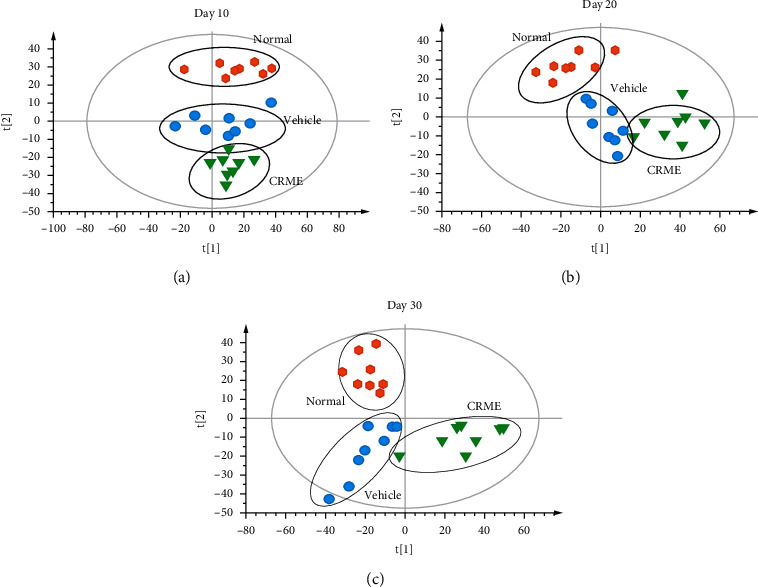
PLS-DA urine score plots on days 10 (a), 20 (b), and 30 (c).

The PLS-DA method was used to evaluate the overall metabolism of the normal control group, CIA model group, and the CRME administration group. Urine sample data from rats in each group were analyzed by PLS-DA on days 10, 20, and 30 (Figure 4). PLS-DA score plots ((A) *R*^2^X = 0.516, *R*^2^Y = 0.929, *Q*^2^_(cum)_ = 0.891; (B) *R*^2^X = 0.521, *R*^2^Y = 0.931, *Q*^2^_(cum)_ = 0.863; (C) *R*^2^X = 0.536, *R*^2^Y = 0.939, *Q*^2^_(cum)_ = 0.791) revealed dynamic metabolite changes in the drug groups on the selected days. The metabolic state of the CRME group differed more from that of the model group with prolonged treatment time (Figure 4), indicating that the metabolic state of CIA rats gradually improved after CRME treatment and that CRME has an obvious therapeutic effect on CIA rats.

### 3.4. Identification of Biomarkers and Analysis of Metabolic Pathways

The PLS-DA analysis value of variable importance in projection (VIP), and the *t*-test method was combined to select compounds with differences (*p* < 0.05). For the urine samples of model rats, 56 differential metabolites (34 in positive, 22 in negative) were confirmed in the final experiment (Table S1). CII significantly disturbs metabolites in rats, leading to a large number of differential metabolites in urine.

**Figure 5:**
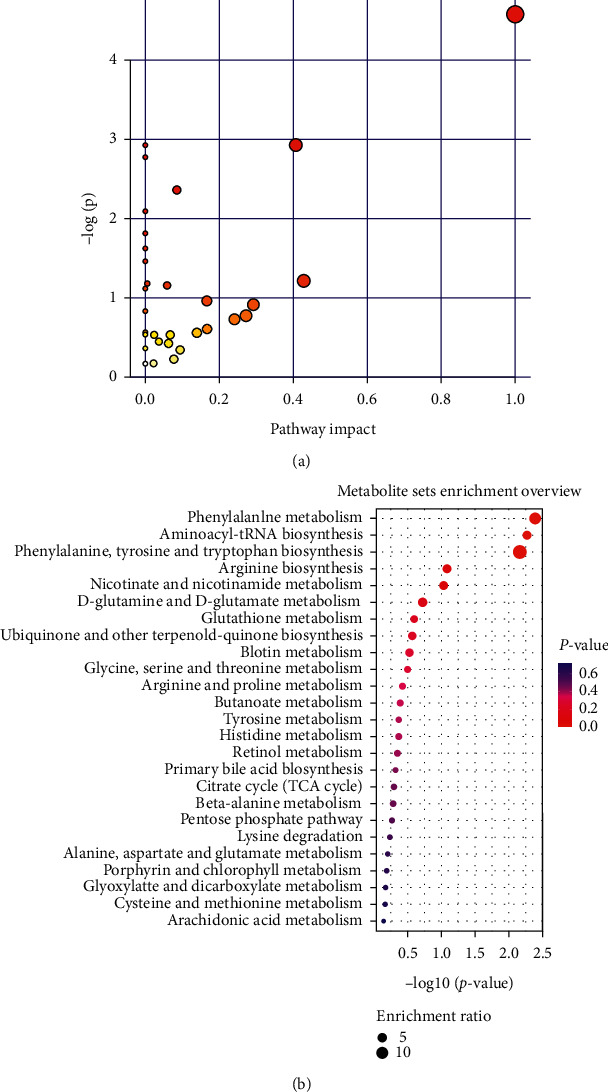
(a) Pathway analysis summary (urine): a: phenylalanine, tyrosine, and tryptophan biosynthesis; b: taurine and hypotaurine metabolism; and c: phenylalanine metabolism. Each point represents one metabolic pathway, and the size of the dot and shades of color represent positive correlations with the impact of the metabolic pathways. (b) Metabolite sets enrichment overview.

**Figure 6:**
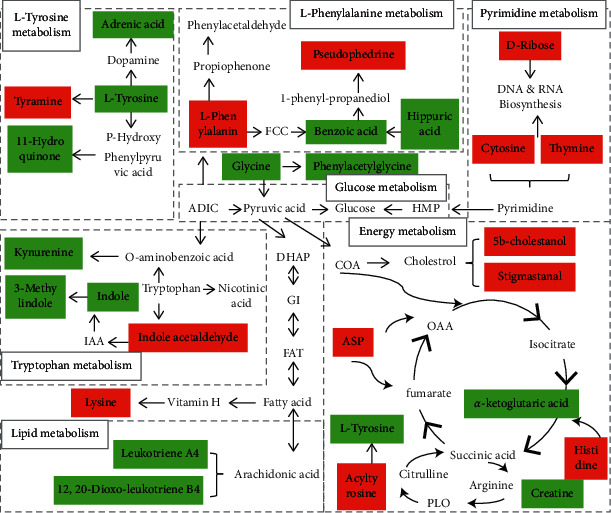
Correlation networks of the perturbed metabolic pathways in collagen-induced arthritis urine models. Red means that the biomarker levels were increased in the model group. Green means that the biomarker levels were decreased in the model group.

We mapped 56 differential metabolites using the KEGG database and obtained 20 associated metabolic pathways, including phenylalanine, tyrosine, and tryptophan biosynthesis (Impact 1), taurine and hypotaurine metabolism (Impact 0.43), phenylalanine metabolism (Impact 0.41), glycine, serine, and threonine metabolism (Impact 0.29), pentose and glucose conversion (Impact 0.27), histidine metabolism (Impact 0.24), tyrosine metabolism (Impact 0.17), riboflavin metabolism (Impact 0.17), and tryptophan metabolism (Impact 0.09). These pathway analysis results are presented graphically (Figure 5) and in a detailed table (Table 2). The link between these pathways and the identified metabolites is illustrated in Figure 6. The metabolic profile data of the urine of rats at 30 days was analyzed to identify the differential changes in metabolites. The effective parts of CRME had different degrees of differential metabolite callbacks using SPSS18.0. Among them, 16 biomarkers have callback effects in urine samples (Figure 1 and Table 1) and are involved in eight metabolic pathways, including tryptophan metabolism, nicotinic acid and nicotinamide metabolism, tyrosine metabolism, phenylalanine metabolism, tricarboxylic acid (TCA cycle), pentose, glucuronic acid conversion, metabolism of cholesterol, and bile acid synthesis.

The pathogenesis of CIA is closely related to the disordered amino acid, glucose, vitamin, and lipid metabolism, which may also be potential metabolic pathway targets for drug therapy.

## 4. Discussion

RA is a disease of unknown etiology that is related to metabolism. According to the theory of TCM, the occurrence of RA is affected by the three major factors of cold (han), damp (shi), and wind (feng), which together affect the stability of the human body environment [3]. There are many drugs clinically used to treat RA, but there is no treatment that is completely effective, nor can RA be completely cured. Therefore, it is important to focus on the mechanism of action of potential drugs for RA treatment. TCM is characterized by multiple components, multiple targets, and high levels of safety. TCM has unique therapeutic advantages for RA, but the mechanism of action is complex and usually involves a wide range of metabolic processes *in vivo*. Clarification of the material basis of TCM effects at the molecular level is a difficult and pressing topic in TCM research. The overall characteristics of metabonomics have led many scholars to apply this approach to the study of TCM metabolites and their pharmacodynamics. Yun et al. studied the interference effect of tetrandrine, in tetrandrine powder, on endogenous metabolites in the urine of CIA rats using the 1H-NMR metabolomics method. They found 23 potential biomarkers of CIA models, which were mainly involved in energy, amino acid, lipid, and intestinal microbial metabolism. The protective effect of pirenoxine on CIA rats may be regulated by these metabolic pathways and potential biomarkers [19]. Li et al. used the UPLC-Q-TOF-MS metabonomic analysis method to study the anti-RA mechanism of clematis triterpene saponins. They found that clematis triterpene saponins can remove citric acid and p-cresol glucose in the serum and urine of CIA rats. Twenty-four biomarkers, including aldehyde and creatinine, return to normal levels following clematis triterpene saponin treatment. This then regulates the overall metabolic pathways of glycerophospholipid metabolism, arachidonic acid metabolism, steroid hormone synthesis, amino acid metabolism, and pantothenic acid and coenzyme A biosynthesis [10]. The effective part of *Caulophyllum robustum* Maxim mainly contains saponins and alkaloids, which are the main material basis of anti-RA of *C. robustum* [8]. Saponin is an important chemical component of traditional Chinese medicine, which has extensive pharmacological effects. Most of the natural saponins exist in the form of sapogenin-linked glycosides. In the process of intestinal metabolism and liver metabolism, most pentacyclic triterpenoid saponins are easily metabolized and converted into aglycones by various enzymes and humoral environments, resulting in biological activity. The bioavailability of pentacyclic triterpenoid saponins is generally low due to poor oral absorption and metabolism [20]. Alkaloids, as an important natural product, are numerous and complex in structure. In recent years, it has been found that alkaloids can be metabolized not only in the liver but also in the intestine, and intestinal flora has a certain metabolic transformation effect on some alkaloids. For the diagnosis of RA, a complex disease, looking for related biomarkers by metabonomics, can improve the accuracy of diagnosis and provide new ideas for the treatment of traditional Chinese medicine [21]. Biomarkers are signal indicators that can reflect the physiological and pathological conditions of the human body and are widely used in disease prediction, diagnosis, and treatment [22]. In a complex disease like RA, finding relevant biomarkers can improve the accuracy of diagnosis and provide new insights into TCM treatment [21]. *C. robostum* Maxim (CRM) has a long history in the treatment of RA in China. However, to date, there have been no studies examining CRM-anti-RA metabolomics. Moreover, there is also a lack of studies on the changes related to metabolic pathways and the identification of biomarkers in RA following treatment with CRME. Therefore, in this study, we established a CII-induced CIA rat model and performed metabolomics analysis of rat urine samples to explore the mechanism of CRME in ameliorating the effects of RA. Key biomarkers were screened and differential metabolites with significant changes in urine samples and their related metabolic pathways were identified.

### 4.1. Amino Acid Metabolism

Phenylalanine hydroxylase converts phenylalanine to tyrosine. Tyrosine is converted to tyramine by amino acid decarboxylase. Tyramine is further metabolized into dopamine, norepinephrine, and other substances with physiological activities. Tyrosine can also be hydrolyzed to fumaric acid, acetoacetic acid, and gentisic acid, into the tricarboxylic acid cyclic. In CIA rats, the levels of hydroquinone and m-cresol decreased and levels of tyrosine, acyl-tyrosine, tyramine, and benzaldehyde increased. The level of m-cresol in the urine of AA rats was increased [23]. Therefore, abnormal tyrosine metabolism will lead to phenylalanine metabolism and energy metabolism disorders, which will affect the overall condition of the body. The level of tyrosine returned to normal in CIA rats after CRME intervention, indicating that CRME could treat RA by regulating tyrosine metabolism.

Tryptophan is the second essential amino acid in animals and plays a key role in the synthesis and metabolism of proteins, enzymes, vitamins, and neurotransmitters [24]. The levels of tryptophan, 5-methoxyamine, and indole acetaldehyde metabolites are increased, and the levels of 2-indolecarboxylic acid, 3-methylindole, indoxyl, kynurenine, and indole-3-methanol are decreased. Tryptophan is deaminated by intestinal flora to produce indoles, which are absorbed into the blood by the intestinal tract and metabolized into indole sulfate by the liver. Indole sulfate has high protein affinity and various cytotoxicity *in vivo*. Tryptophan plays an important role in regulating T cell-mediated autoimmune disease [25], and it can affect T cell proliferation in patients with RA by regulating the IDO/TTS tryptophan metabolism pathway. It is also possible to clear autoreactive T cell-mediated chronic immune inflammation. Kynurenine is an important metabolite in the tryptophan metabolism pathway. The ratio of kynurenine to tryptophan is an immune status index *in vivo* and is significantly higher in the blood of patients with RA than in those without RA [26]. The levels of 2-indolecarboxylic acid and indole phenol returned to normal after the intervention with CRME, suggesting that CRME can alleviate the inflammatory symptoms of RA by regulating the response of tryptophan in the intestinal flora.

The histidine content in the urine of CIA rats is high, and the leucine to histidine ratio can be a potential biomarker for clinical osteoarthritis. Early studies have found that histidine metabolism is elevated in patients with RA, but the specific pathological mechanism remains unclear. This is consistent with our findings that histamine metabolism disorder is also a pathological feature of RA.

### 4.2. Glutathione Metabolism

Glutathione is a tripeptide consisting of glutamic acid, cysteine, and glycine and contains *γ*-amide bonds and mercapto groups. Glutathione is found in almost every cell of the body and helps the immune system function [27]. Glycine receptors are distributed in the cell membranes of monocytes, macrophages, neutrophils, T-lymphocytes, and vascular endothelial cells and participate in immune responses and inflammation. Liu et al. [28] also found that acute gouty arthritis can cause disorders of the glutathione metabolism pathway. In the field of medicine, it has been reported that glutathione can treat eye diseases such as cataract, alleviate hepatitis, and protect the liver [29] and has antioxidant [27] and anti-HIV effects [30]. Glutathione is involved in apoptosis, signal transduction, and regulation of nitric oxide signaling pathways and induces oxidative stress responses in cells [31]. The levels of methionine, glycine, and phenylacetyl glycine in urine samples from CIA rats were decreased, indicating that glutathione metabolism was disordered in CIA rats.

### 4.3. Energy Metabolism

The tricarboxylic acid cycle is the final energy-producing carbohydrate, lipid, and amino acid metabolic pathway and is the hub of carbohydrate, lipid, and amino acid metabolism [32]. The tricarboxylic acid cycle also provides small molecular precursors for other synthetic metabolic processes. For example, oxaloacetic acid is the precursor of aspartic acid, *α*-ketoglutaric acid is the precursor for glutamic acid synthesis, and some amino acids can be converted to sugar through this pathway. Creatine and its phosphorylated form are intermediates of energy metabolism, and a decrease in these molecules may be related to energy demand. Since phosphocreatine has a direct role in cellular energy transport, creatine can supply energy to the muscles in the form of stored creatine phosphate. As energy requirements increase, muscles can rapidly resynthesize ADP into ATP through creatine and creatine kinase systems. The levels of glutaric acid and *α* -ketoglutaric acid in urine samples from CIA rats decreased, indicating disordered energy metabolism in the model rats. Abnormal energy metabolism was found in AA rats, and *α*-ketoglutaric acid was used as a biomarker of RA.

### 4.4. Vitamin Metabolism

The nicotinic acid, retinoic acid, methyl nicotinamide, coenzyme Q9, and *α*-CEHC levels in urine samples from CIA rats increased, while N1-methyl-4-pyridone-3-formamide levels decreased. Retinoic acid and coenzyme Q9 are vitamin A and vitamin H molecules, respectively. Nicotinic acid, methyl nicotinamide, and N1-methyl-4-pyridone-3-formamide are nicotinamide components and belong to the B group vitamins. B vitamins are water-soluble and can promote the process of biological oxidation and metabolism. Tryptophan provides pyridine nucleotides to the liver through the nicotinamide adenine dinucleotide pathway to produce nicotinamide, which is converted to N-methyl nicotinamide by N-methyltransferase and then to N1-methyl-4-pyridinone-3-formamide by aldehyde oxidase. *a*-CEHC can reflect vitamin E metabolism status in the body. Vitamin E is a fat-soluble antioxidant and is an important component of both cell membranes and lipoproteins. When vitamin E is lacking, free radicals are produced in the body that not only damage the structure and function of the cell membrane but also cause the immune system to decline and lead to abnormal metabolism. Therefore, vitamins are important for maintaining and regulating normal metabolism. The change in vitamins in the urine of CIA rats indicates that RA will cause the immune system to decline and affect normal metabolic dysfunction. It can be inferred that RA may lead to decreased immune system activity and disordered metabolic function according. In CIA rats, the levels of *α*-CEHC and N1-methyl-4-pyridine-3-formamide returned to normal after CRME intervention, indicating that CRME can regulate vitamin E and vitamin B3 metabolic pathways and regulate the disease state of RA through vitamins.

### 4.5. Lipid Metabolism

The levels of 17-*β*-estradiol-3-glucuronoside, cholestanol, trihydroxycholane, stigmasterol, 7-hydroxy-3-oxycholic acid, and phosphatidylserine, products of lipid metabolism, increased. The levels of chenodeoxycholic acid, deoxycortisol, leukotriene A4, leukotriene B4, 7-ketodeoxycholic acid, 7-sulfonylcholic acid, and 14-hydroxy 22 carbon hexanoic acid decreased. From the identified metabolites, lipid metabolic disorders mainly focus on bile acid synthesis, steroid hormone biosynthesis, and unsaturated fatty acid metabolism. Moreover, estradiol combines with glucuronic acid in the uronic acid pathway to form active 17-*β*-estradiol-3-glucuronide.

Cholesterol, also known as dihydrocholesterol, is a sterol. Trihydroxycholestane induces apoptosis in malignant glioma cells by activating internal and external apoptotic pathways, and Bcl-2 family proteins play an important regulatory role in this process. Stigmasterol has strong anti-inflammatory effects and directly used as an anti-inflammatory drug. Cholesterol is an important component of cell membranes and plasma lipoprotein and is the precursor of many important steroids including bile acid, adrenocortical hormone, estrogen, androgen, and vitamin D3. Most cholesterol can be converted into bile acid, and the imbalance of cholesterol metabolism can have adverse effects on the body.

Leukotriene A4 (LTA4) and leukotriene B4 (LTB4) are bioactive lipid mediators produced by 5-lipoxygenase metabolizing arachidonic acid. Leukotrienes are chemical mediators in some allergic, inflammatory, and cardiovascular diseases and play an important role in RA and gout. While the leukotriene content in the body should be significantly increased with inflammation, we found that it was decreased in CIA rats. The specific reasons for this require further study. After CRME intervention, the levels of 17-*β*-estradiol-3-glucuronoside, cholestanol, trihydroxycholestane, stigmasterol, and 7-hydroxy-3-oxycholic acid returned to normal, indicating that CRME can treat RA by regulating steroid hormone and bile acid metabolism.

### 4.6. Other Metabolism

Pyridinol has emerged as a biomarker of bone metabolism in recent years [33]. After an osteoporotic fracture, deoxypyridinoline shows a trend to increase in the urine. The observed decrease of the urine pyridinol content in CIA rats represents abnormal bone metabolism *in vivo* [34].

In summary, amino acids play an important role in the pathogenesis of RA. The amino acid metabolism changes observed in CIA rats may be the main cause of immune activation in the pathogenesis of RA. After intervention with CRME, 16 metabolites including benzoic acid, hippuric acid, hydroquinone, tyrosine, and indophenol returned to normal levels, indicating that CRME can treat RA by regulating amino acid, energy, and lipid metabolism.

Fifty-six potential biomarkers related to CIA and 20 metabolic pathways disturbed by CIA were preliminarily identified. Based on these results, the pathogenesis of RA and metabolic disorders was proposed. We describe that phenylalanine, tyrosine, and tryptophan biosynthesis (Impact 1), taurine and subtaurine metabolism (Impact 0.43), phenylalanine metabolism (Impact 0.41), glycine, serine, and threonine metabolism (Impact 0.29), pentose and glucose conversion (Impact 0.27), histidine metabolism (Impact 0.24), tyrosine metabolism (Impact 0.17), riboflavin metabolism (Impact 0.17), and tryptophan metabolism (Impact 0.09) were significantly disturbed. After the CRME intervention CIA model rats, 16 kinds of differential metabolites were regulated to the normal state, and the disordered metabolic spectrum returned to normal levels. The levels of 2-indolecarboxylic acid, hydroquinone, benzoic acid, and hippuric acid were significantly upregulated, while the levels of 17-*β*-estradiol-3-glucuronoside, cholestanol, 3-hydroxycholestane, stigmasterol, tyrosine, D-ribose, N1-methyl-4-pyridone-3-formamide, 2-diaminobutyric acid, monoisobutylphthalic acid, *α*-CEHC, and 7-hydroxy-oxycholic acid were significantly downregulated. Together, these results show the urine metabolic spectrum reveals that the pathogenesis of CIA in rats is closely related to the amino acid, energy, and lipid metabolism. CRME has a certain regulatory effect on disorders of amino acid, vitamin, and lipid metabolism caused by CIA. The metabonomics results suggest that regulating amino acid, vitamin, energy, and lipid metabolism may be a potential target pathway for CRME in the treatment of RA.

## 5. Conclusions

This study employed an innovative approach to look for biomarkers and metabolic pathways related to the pathogenesis of RA by analyzing urine samples. On the basis of traditional metabonomics research, the potential biomarkers found were quantified using LC-MS, and the quantitative indexes were given. Commonly used clinical therapeutic drugs were used for methodological verification, and a new metabonomics method was developed to study the overall action mechanism of RA therapeutic drugs. The established metabolomics model was used to study the mechanism of CRME action against RA. The 56 metabolites in the urines might be associated with the treatment response of *C. robustum*, of which, 16 metabolites were found to be obviously changed after C. robustum treatment. These endogenous metabolites such as tyrosine, alpha-CEHC, and 2-indolecarboxylic acid were assumed as potential biomarkers of RA, which are connected with various metabolic pathways such as amino acid, vitamin, energy, and lipid metabolism. The anti-RA effect of *Caulophyllum robustum* Maxim is exerted by regulating pathways of amino acid, vitamin, energy, and lipid metabolism. This is the first time that a metabolomic approach has been adopted to account for the anti-RA action of *Caulophyllum robustum* Maxim. The method of this study reflects the “holistic” characteristics of TCM and is expected to provide strong technical support for the study of the mechanism of CRME in the treatment of RA.

## Figures and Tables

**Table 1:** The identification of potential biomarkers and their metabolic pathways.

No.	Rt (min)	m/*z*	Formula	Description	Metabolic pathways	Trend
1	0.90	153.0662	C_7_H_8_N_2_O_2_	N1-methyl-4-pyridone-3-carboxamide	Vitamin metabolism	↓
2	11.93	101.0712	C_4_H_10_N_2_O_2_	2,4-Diaminobutyric acid	Amino acid neurotransmitter metabolism	↑
3	12.37	413.2715	C_24_H_38_O_4_	7-Hydroxy-3-oxocholanoic acid	Bile acid metabolism	↑
4	11.68	301.1428	C_16_H_22_O_4_	alpha-CEHC	Vitamin metabolism	↑
5	4.95	160.0403	C_9_H_7_NO_2_	2-Indolecarboxylic acid	Tryptophan metabolism	↓
6	5.94	429.1970	C_24_H_32_O_8_	17-beta-estradiol-3-glucuronide	Pentose and glucuronate interconversions	↑
7	12.79	433.3698	C_27_H_48_O	5b-Cholestanol	Cholesterol metabolism	↑
8	4.32	180.0670	C_9_H_9_NO_3_	Hippuric acid	Phenylalanine metabolism	↓
9	0.90	151.0621	C_5_H_10_O_5_	D-Ribose	Pentose phosphate pathway	↑
10	4.32	105.0350	C_7_H_6_O_2_	Benzoic acid	Phenylalanine metabolism	↓
11	11.69	205.0868	C_12_H_14_O_4_	Monoisobutyl phthalic acid	Energy metabolism	↑
12	12.62	419.3532	C_27_H_48_O_3_	5-b-cholestane-3a,7a,12a-triol	Cholesterol metabolism	↑
13	3.66	156.0408	C _8_H_7_NO	Indoxyl	Tryptophan metabolism	↓
14	164.07	1.2379	C_9_H_11_NO_3_	L-Tyrosine	Tyrosine metabolism	↓
15	461.40	2.04602	C_29_H_52_O	Stigmastanol	Cholesterol metabolism	↑
16	109.03	3.07623	C_6_H_6_O_2_	11-Hydroquinone	Tyrosine metabolism	↓

**Table 2:** Rat urine metabolic pathway enrichment table.

No.	Pathway name	Match status	*p*	−log(p)	Impact
1	Phenylalanine, tyrosine and tryptophan biosynthesis	2/4	0.0102	4.5829	1
2	Taurine and hypotaurine metabolism	1/8	0.2959	1.2178	0.429
3	Phenylalanine metabolism	2/9	0.0534	2.9294	0.407
4	Glycine, serine, and threonine metabolism	2/32	0.4017	0.9112	0.292
5	Pentose and glucuronate interconversions	1/14	0.4595	0.7776	0.273
6	Histidine metabolism	1/15	0.4829	0.7280	0.242
7	Tyrosine metabolism	2/42	0.5451	0.6068	0.167
8	Riboflavin metabolism	1/11	0.3830	0.9597	0.167
9	Tryptophan metabolism	4/41	0.0943	2.3615	0.086
10	Primary bile acid biosynthesis	3/46	0.3142	1.1576	0.059
11	Glutathione metabolism	2/26	0.3067	1.1818	0.006

## Data Availability

Baseline filter, peak identification, integration, retention time correction, peak alignment, and normalization were performed by the metabolomics processing software of the instrument Progenesis QI (Waters Corporation) using raw data to obtain a data matrix with retention time, mass-to-charge ratio, and peak intensity. The normalized urine sample data matrix was imported into the SIMCA-P + 14.0 software package (Umetrics, Umea, Sweden) for unsupervised principal components analysis (PCA) to observe the overall sample distribution and the stability of the analysis process. Then, the supervised (orthogonal) partial least square method, (O)PLS-DA, was used to distinguish overall metabolic profile differences and identify metabolite differences between groups. Variables with variable important in projection (VIP) scores greater than 1 were considered differential variables. To prevent the model overfitting, the quality of the model was investigated using seven interactive verification cycles and 200 response sequencing tests. Screening the metabolite differences between groups (VIP > 1, *p* < 0.05, Fold Change (FC) > 1.2) was achieved using multidimensional analysis of PLS-DA and single-dimensional analysis (Student's *t*-test) methods. Differential metabolites were selected for qualitative analysis using http://www.hmdb.ca/and self-built databases.

## Abbreviations

RA: Rheumatoid arthritis
TCM: Traditional Chinese medicine
CRM: *Caulophyllum robustum* Maxim
CRME: *Caulophyllum robustum* Maxim extract
UPLC-Q-TOF/MS: Ultra-performance Liquid Chromatography-Quadrupole-Time-of-Flight/Mass Spectrometry
NMR: Nuclear magnetic resonance
LC-MS: Liquid chromatography-mass spectrometry
GC-MS: Gas chromatography-mass spectrometry
PCA: Principal component analysis
PLS-DA: Partial least squares discrimination analysis
OPLS-DA: Orthogonal partial least squares discriminant analysis
VIP: Variable importance for the prediction
CFA: Complete Freund's adjuvant
IFA: Incomplete Freund's adjuvant
BPI: Basic peak ion chromatogram
S1P: Sphingosine-1-Phosphate.

## Supplementary Materials

Supplementary Materials For the urine samples of model rats, 56 differential metabolites (34 in positive, 22 in negative) were confirmed in the final experiment (Table S1). BPI chromatograms in positive and negative ion modes are shown in the supplementary material (Figure S1).
