# Supplementary material for: Metabolomic Analysis of the Urine from Rats with Collagen-Induced Arthritis with the Effective Part of Caulophyllum robustum Maxim
Source: Evid Based Complement Alternat Med. 2021 May 26;2021:5580341. doi: 10.1155/2021/5580341 (PMC8189773; doi:10.1155/2021/5580341)
Supplement: Supplementary Materials — For the urine samples of model rats, 56 differential metabolites (34 in positive, 22 in negative) were confirmed in the final experiment (Table S1). BPI chromatograms in positive and negative ion modes are shown in the supplementary material (Figure S1). [file 5580341.f1.docx]

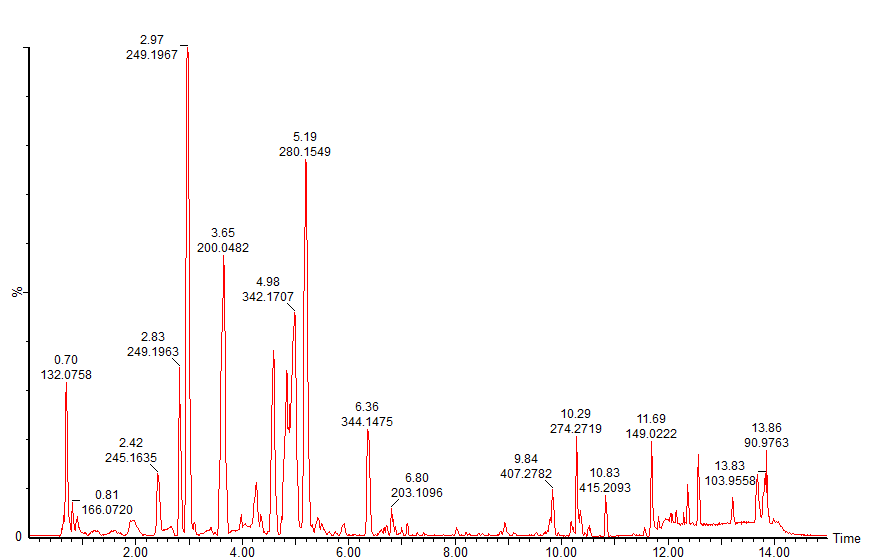

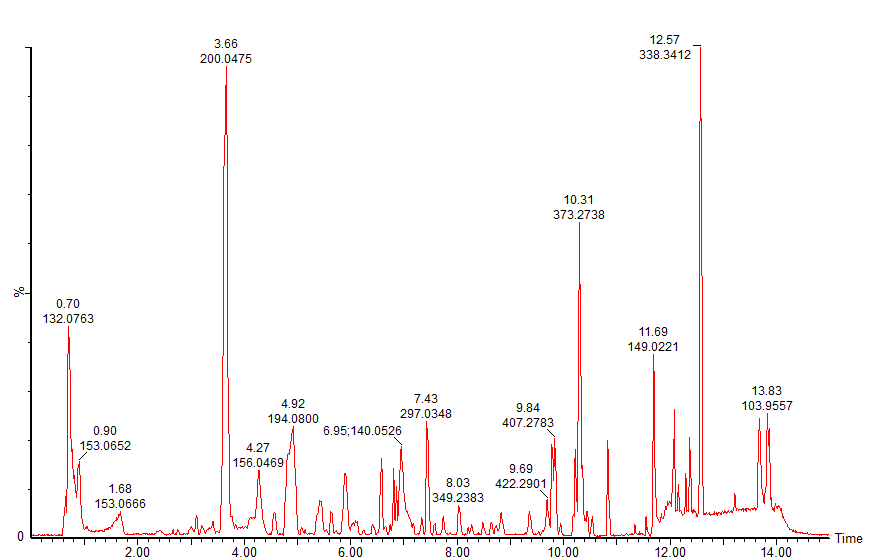


+ESIB

+ESIA


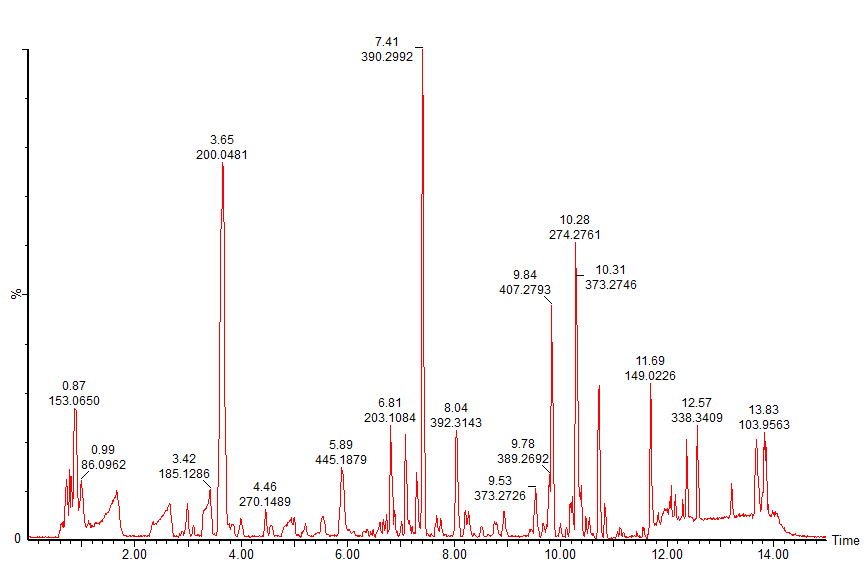


+ESIC


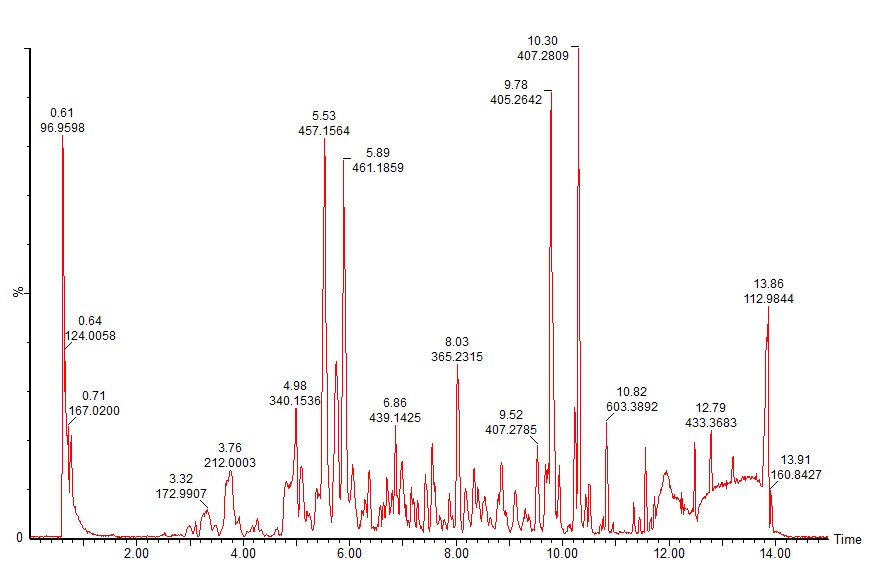


-ESIB

-ESIA


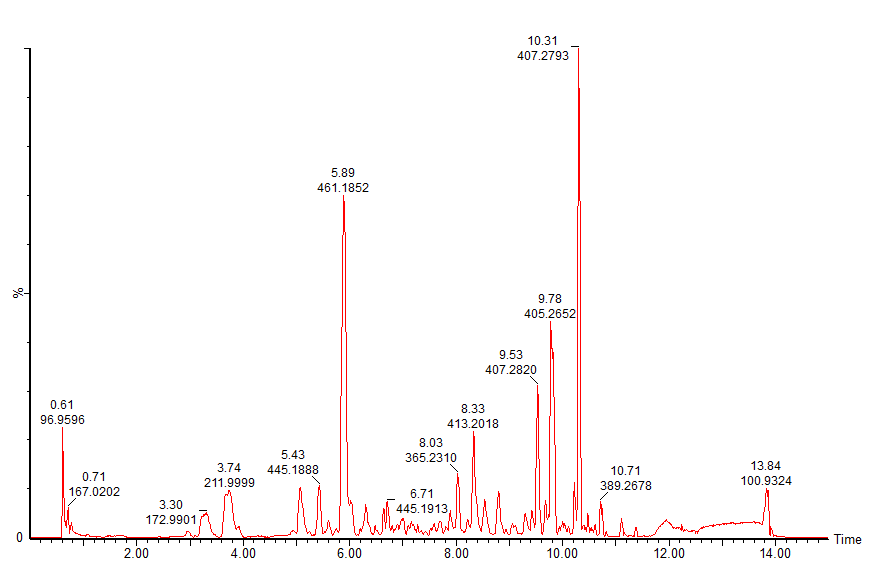


-ESIC

FigS1 ESI (+) and ESI (-) BPI chromatogram profile of metabolites in rat urine (A,B,C) by UPLC-Q-TOF/MS

(Normal:A; Vehicle: B;CRME:C)

| **NO.**  Tab.S1 Identities of differential metabolites between model and normal rats in urine | **Compound ID** | **ion mode** | **Rt**  **min** | **m/z** | **VIP value** | **Formula** | **Description** | **[KEGG](https://cn.bing.com/dict/search?q=KEGG&FORM=BDVSP6&mkt=zh-cn) [metabolic](https://cn.bing.com/dict/search?q=metabolic&FORM=BDVSP6&mkt=zh-cn) [pathways](https://cn.bing.com/dict/search?q=pathways&FORM=BDVSP6&mkt=zh-cn)** | **Trend** |
| --- | --- | --- | --- | --- | --- | --- | --- | --- | --- |
| 1 | HMDB01131 | - | 13.03 | 151.9964 | 1.0301 | C_4_H_5_NO_4_ | Iminoaspartic acid | [Aspartic](https://cn.bing.com/dict/search?q=aspartic&FORM=BDVSP6&mkt=zh-cn) [acid](https://cn.bing.com/dict/search?q=acid&FORM=BDVSP6&mkt=zh-cn) [metabolism](https://cn.bing.com/dict/search?q=metabolism&FORM=BDVSP6&mkt=zh-cn) | ↑ |
| 2 | HMDB04072 | - | 5.58 | 165.0555 | 1.3066 | C_8_H_8_O | 4-Hydroxystyrene | Tyrosine [metabolism](https://cn.bing.com/dict/search?q=metabolism&FORM=BDVSP6&mkt=zh-cn) | ↓ |
| 3 | HMDB02434 | - | 2.79 | 109.0294 | 3.07623 | C_6_H_6_O_2_ | 11-Hydroquinone | Tyrosine [metabolism](https://cn.bing.com/dict/search?q=metabolism&FORM=BDVSP6&mkt=zh-cn) | ↓ |
| 4 | HMDB06115 | - | 8.76 | 257.0818 | 1.1435 | C_7_H_6_O | Benzaldehyde | Tyrosine [metabolism](https://cn.bing.com/dict/search?q=metabolism&FORM=BDVSP6&mkt=zh-cn) | ↑ |
| 5 | HMDB00866 | - | 5.43 | 224.0959 | 12.5017 | C_11_H_13_NO_4_ | N-Acetyl-L-tyrosine | Tyrosine [metabolism](https://cn.bing.com/dict/search?q=metabolism&FORM=BDVSP6&mkt=zh-cn) | ↑ |
| 6 | HMDB02285 | - | 4.95 | 160.0403 | 1.60283 | C_9_H_7_NO_2_ | 2-Indolecarboxylic acid | [Tryptophan](https://cn.bing.com/dict/search?q=tryptophan&FORM=BDVSP6&mkt=zh-cn) [metabolism](https://cn.bing.com/dict/search?q=metabolism&FORM=BDVSP6&mkt=zh-cn) | ↓ |
| 7 | HMDB01896 | - | 6.78 | 427.1842 | 1.0155 | C_11_H_13_NO_2_ | 5-Methoxytryptophol | [Tryptophan](https://cn.bing.com/dict/search?q=tryptophan&FORM=BDVSP6&mkt=zh-cn) [metabolism](https://cn.bing.com/dict/search?q=metabolism&FORM=BDVSP6&mkt=zh-cn) | ↑ |
| 8 | HMDB00466 | - | 6.64 | 130.0662 | 1.4332 | C_9_H_9_N | 3-Methylindole | [Tryptophan](https://cn.bing.com/dict/search?q=tryptophan&FORM=BDVSP6&mkt=zh-cn) [metabolism](https://cn.bing.com/dict/search?q=metabolism&FORM=BDVSP6&mkt=zh-cn) | ↓ |
| 9 | HMDB00620 | - | 0.73 | 175.0247 | 1.0466 | C_5_H_6_O_4_ | Glutaconic acid | [Taurine](https://cn.bing.com/dict/search?q=Taurine&FORM=BDVSP6&mkt=zh-cn) [and](https://cn.bing.com/dict/search?q=and&FORM=BDVSP6&mkt=zh-cn) [hypotaurine](https://cn.bing.com/dict/search?q=hypotaurine&FORM=BDVSP6&mkt=zh-cn) [metabolism](https://cn.bing.com/dict/search?q=metabolism&FORM=BDVSP6&mkt=zh-cn) | ↓ |
| 10 | HMDB00208 | - | 0.71 | 191.0217 | 3.1493 | C_5_H_6_O_5_ | Oxoglutaric acid | [Taurine](https://cn.bing.com/dict/search?q=Taurine&FORM=BDVSP6&mkt=zh-cn) [and](https://cn.bing.com/dict/search?q=and&FORM=BDVSP6&mkt=zh-cn) [hypotaurine](https://cn.bing.com/dict/search?q=hypotaurine&FORM=BDVSP6&mkt=zh-cn) [metabolism](https://cn.bing.com/dict/search?q=metabolism&FORM=BDVSP6&mkt=zh-cn) | ↓ |
| 11 | HMDB06224 | - | 5.94 | 429.1970 | 2.8946 | C_24_H_32_O_8_ | 17-beta-Estradiol-3-glucuronide | Pentose and glucuronate interconversions | ↑ |
| 12 | HMDB02577 | - | 8.39 | 583.3171 | 2.4378 | C_30_H_48_O_11_ | Cholic acid glucuronide | Pentose and glucuronate interconversions | ↓ |
| 13 | HMDB00015 | - | 10.43 | 345.2080 | 5.6690 | C_21_H_30_O_4_ | Cortexolon | [Steroid](https://cn.bing.com/dict/search?q=steroid&FORM=BDVSP6&mkt=zh-cn) [metabolism](https://cn.bing.com/dict/search?q=metabolism&FORM=BDVSP6&mkt=zh-cn) | ↓ |
| 14 | HMDB01569 | - | 12.79 | 433.3698 | 3.2947 | C_27_H_48_O | 5b-Cholestanol | [Cholesterol](https://cn.bing.com/dict/search?q=cholesterol&FORM=BDVSP6&mkt=zh-cn) [metabolism](https://cn.bing.com/dict/search?q=metabolism&FORM=BDVSP6&mkt=zh-cn) | ↑ |
| 15 | HMDB01457 | - | 12.62 | 419.3532 | 1.11754 | C_27_H_48_O_3_ | 5-b-Cholestane-3a,7a,12a-triol | [Cholesterol](https://cn.bing.com/dict/search?q=cholesterol&FORM=BDVSP6&mkt=zh-cn) [metabolism](https://cn.bing.com/dict/search?q=metabolism&FORM=BDVSP6&mkt=zh-cn) | ↑ |
| 16 | HMDB00494 | - | 13.19 | 461.4011 | 2.04602 | C_29_H_52_O | Stigmastanol | [Cholesterol](https://cn.bing.com/dict/search?q=cholesterol&FORM=BDVSP6&mkt=zh-cn) [metabolism](https://cn.bing.com/dict/search?q=metabolism&FORM=BDVSP6&mkt=zh-cn) | ↑ |
| 17 | HMDB02421 | - | 8.06 | 487.2381 | 1.5031 | C_24_H_40_O_8_S | 7-Sulfocholic acid | [Cholesterol](https://cn.bing.com/dict/search?q=cholesterol&FORM=BDVSP6&mkt=zh-cn) [metabolism](https://cn.bing.com/dict/search?q=metabolism&FORM=BDVSP6&mkt=zh-cn) | ↓ |
| 18 | HMDB60044 | - | 8.83 | 401.2332 | 2.9863 | C_23_H_32_O_3_ | 14-HDoHE | [Fatty](https://cn.bing.com/dict/search?q=fatty&FORM=BDVSP6&mkt=zh-cn) [acid](https://cn.bing.com/dict/search?q=acid&FORM=BDVSP6&mkt=zh-cn) [metabolism](https://cn.bing.com/dict/search?q=metabolism&FORM=BDVSP6&mkt=zh-cn) | ↓ |
| 19 | HMDB02522 | - | 10.25 | 471.2437 | 2.2808 | C_24_H_40_O_7_S | Chenodeoxycholic acid sulfate | [Fatty](https://cn.bing.com/dict/search?q=fatty&FORM=BDVSP6&mkt=zh-cn) [acid](https://cn.bing.com/dict/search?q=acid&FORM=BDVSP6&mkt=zh-cn) [metabolism](https://cn.bing.com/dict/search?q=metabolism&FORM=BDVSP6&mkt=zh-cn) | ↓ |
| 20 | HMDB60094 | - | 7.61 | 329.1754 | 1.3813 | C_20_H_28_O_5_ | 12,20-Dioxo-leukotriene B4 | [Arachidonic](https://cn.bing.com/dict/search?q=arachidonic&FORM=BDVSP6&mkt=zh-cn) [acid](https://cn.bing.com/dict/search?q=acid&FORM=BDVSP6&mkt=zh-cn) | ↓ |
| 21 | HMDB01337 | - | 8.73 | 363.2175 | 2.9078 | C_20_H_30_O_3_ | Leukotriene A4 | [Arachidonic](https://cn.bing.com/dict/search?q=arachidonic&FORM=BDVSP6&mkt=zh-cn) [acid](https://cn.bing.com/dict/search?q=acid&FORM=BDVSP6&mkt=zh-cn) | ↓ |
| 22 | HMDB00851 | - | 8.12 | 427.1801 | 1.2233 | C_18_H_28_N_4_O_8_ | Pyridinoline | [Bone](https://cn.bing.com/dict/search?q=Bone&FORM=BDVSP6&mkt=zh-cn) [metabolism](https://cn.bing.com/dict/search?q=Metabolism&FORM=BDVSP6&mkt=zh-cn) | ↓ |
| 1 | HMDB00123 | + | 4.91 | 76.0400 | 3.7449 | C_2_H_5_NO_2_ | Glycine | [Glycine](https://cn.bing.com/dict/search?q=glycine&FORM=BDVSP6&mkt=zh-cn) [metabolism](https://cn.bing.com/dict/search?q=metabolism&FORM=BDVSP6&mkt=zh-cn) | ↓ |
| 2 | HMDB00821 | + | 4.91 | 194.0827 | 4.4962 | C_10_H_11_NO_3_ | Phenylacetylglycin | [Glycine](https://cn.bing.com/dict/search?q=glycine&FORM=BDVSP6&mkt=zh-cn) [metabolism](https://cn.bing.com/dict/search?q=metabolism&FORM=BDVSP6&mkt=zh-cn) | ↓ |
| 3 | HMDB00064 | + | 0.71 | 114.0672 | 1.0883 | C_4_H_9_N_3_O_2_ | Creatine | [Glycine](https://cn.bing.com/dict/search?q=glycine&FORM=BDVSP6&mkt=zh-cn) [metabolism](https://cn.bing.com/dict/search?q=metabolism&FORM=BDVSP6&mkt=zh-cn) | ↓ |
| 4 | HMDB00715 | + | 3.85 | 190.0504 | 1.3240 | C_10_H_7_NO_3_ | Kynurenic acid | [Tryptophan](https://cn.bing.com/dict/search?q=tryptophan&FORM=BDVSP6&mkt=zh-cn) [metabolism](https://cn.bing.com/dict/search?q=metabolism&FORM=BDVSP6&mkt=zh-cn) | ↓ |
| 5 | HMDB05785 | + | 5.08 | 130.0653 | 1.6852 | C_9_H_9_NO | Indole-3-carbinol | [Tryptophan](https://cn.bing.com/dict/search?q=tryptophan&FORM=BDVSP6&mkt=zh-cn) [metabolism](https://cn.bing.com/dict/search?q=metabolism&FORM=BDVSP6&mkt=zh-cn) | ↓ |
| 6 | HMDB01190 | + | 6.96 | 160.0759 | 1.2447 | C_10_H_9_NO | Indoleacetaldehyde | [Tryptophan](https://cn.bing.com/dict/search?q=tryptophan&FORM=BDVSP6&mkt=zh-cn) [metabolism](https://cn.bing.com/dict/search?q=metabolism&FORM=BDVSP6&mkt=zh-cn) | ↑ |
| 7 | HMDB04094 | + | 3.66 | 156.0408 | 3.1630 | C _8_H_7_NO | Indoxyl | [Tryptophan](https://cn.bing.com/dict/search?q=tryptophan&FORM=BDVSP6&mkt=zh-cn) [metabolism](https://cn.bing.com/dict/search?q=metabolism&FORM=BDVSP6&mkt=zh-cn) | ↓ |
| 8 | HMDB00158 | + | 5.45 | 164.0711 | 1.2379 | C_9_H_11_NO_3_ | 1. Tyrosin | Tyrosine [metabolism](https://cn.bing.com/dict/search?q=metabolism&FORM=BDVSP6&mkt=zh-cn) | ↓ |
| 9 | HMDB00306 | + | 2.66 | 120.0811 | 1.2206 | C_8_H_11_NO | Tyramine | Tyrosine [metabolism](https://cn.bing.com/dict/search?q=metabolism&FORM=BDVSP6&mkt=zh-cn) | ↑ |
| 10 | HMDB00714 | + | 4.32 | 180.0670 | 3.6622 | C_9_H_9_NO_3_ | Hippuric acid | [Phenylalanine](https://cn.bing.com/dict/search?q=Phenylalanine&FORM=BDVSP6&mkt=zh-cn) [metabolism](https://cn.bing.com/dict/search?q=metabolism&FORM=BDVSP6&mkt=zh-cn) | ↓ |
| 11 | HMDB01870 | + | 4.32 | 105.0350 | 6.5706 | C_7_H_6_O_2_ | Benzoic acid | [Phenylalanine](https://cn.bing.com/dict/search?q=Phenylalanine&FORM=BDVSP6&mkt=zh-cn) [metabolism](https://cn.bing.com/dict/search?q=metabolism&FORM=BDVSP6&mkt=zh-cn) | ↓ |
| 12 | HMDB00159 | + | 2.66 | 166.0864 | 1.1666 | C_9_H_11_NO_2_ | 1. Phenylalanine | [Phenylalanine](https://cn.bing.com/dict/search?q=Phenylalanine&FORM=BDVSP6&mkt=zh-cn) [metabolism](https://cn.bing.com/dict/search?q=metabolism&FORM=BDVSP6&mkt=zh-cn) | ↑ |
| 13 | HMDB01943 | + | 3.76 | 148.1128 | 1.1480 | C_10_H_15_NO | Pseudoephedrine | [Phenylalanine](https://cn.bing.com/dict/search?q=Phenylalanine&FORM=BDVSP6&mkt=zh-cn) [metabolism](https://cn.bing.com/dict/search?q=metabolism&FORM=BDVSP6&mkt=zh-cn) | ↑ |
| 14 | HMDB00434 | + | 5.20 | 197.0830 | 1.8136 | C_10_H_12_O_4_ | Homoveratric acid | [Phenylalanine](https://cn.bing.com/dict/search?q=Phenylalanine&FORM=BDVSP6&mkt=zh-cn) [metabolism](https://cn.bing.com/dict/search?q=metabolism&FORM=BDVSP6&mkt=zh-cn) | ↑ |
| 15 | HMDB00182 | + | 0.61 | 147.1129 | 1.2911 | C_6_H_14_N_2_O_2_ | 1. Lysine | [Lysine](https://cn.bing.com/dict/search?q=Lysine&FORM=BDVSP6&mkt=zh-cn) [metabolism](https://cn.bing.com/dict/search?q=metabolism&FORM=BDVSP6&mkt=zh-cn) | ↑ |
| 16 | HMDB00070 | + | 0.72 | 130.0866 | 4.7519 | C_6_H_11_NO_2_ | Pipecolic acid | [Lysine](https://cn.bing.com/dict/search?q=Lysine&FORM=BDVSP6&mkt=zh-cn) [metabolism](https://cn.bing.com/dict/search?q=metabolism&FORM=BDVSP6&mkt=zh-cn) | ↓ |
| 17 | HMDB00679 | + | 0.71 | 212.1037 | 2.7624 | C_7_H_15_N_3_O_3_ | Homocitrulline | Citrulline metabolism | ↓ |
| 18 | HMDB00214 | + | 4.08 | 265.1897 | 2.8639 | C_5_H_12_N_2_O_2_ | Ornithi | [Arginine](https://cn.bing.com/dict/search?q=Arginine&FORM=BDVSP6&mkt=zh-cn) [and](https://cn.bing.com/dict/search?q=and&FORM=BDVSP6&mkt=zh-cn) [Proline](https://cn.bing.com/dict/search?q=Proline&FORM=BDVSP6&mkt=zh-cn) [metabolism](https://cn.bing.com/dict/search?q=metabolism&FORM=BDVSP6&mkt=zh-cn) | ↑ |
| 19 | HMDB00177 | + | 0.62 | 156.0769 | 1.0575 | C_6_H_9_N_3_O_2_ | 1. Histidine | [Histidine](https://cn.bing.com/dict/search?q=histidine&FORM=BDVSP6&mkt=zh-cn) [metabolism](https://cn.bing.com/dict/search?q=metabolism&FORM=BDVSP6&mkt=zh-cn) | ↑ |
| 20 | HMDB00696 | + | 4.39 | 172.0430 | 1.1712 | C_5_H_11_NO_2_S | 1. Methionine | [Glutathione](https://cn.bing.com/dict/search?q=glutathione&FORM=BDVSP6&mkt=zh-cn) [metabolism](https://cn.bing.com/dict/search?q=metabolism&FORM=BDVSP6&mkt=zh-cn) | ↓ |
| 21 | HMDB00283 | + | 0.90 | 151.0621 | 2.2789 | C_5_H_10_O_5_ | D-Ribose | Pentose phosphate pathway | ↑ |
| 22 | HMDB02056 | + | 11.69 | 205.0868 | 2.26043 | C_12_H_14_O_4_ | Monoisobutyl phthalic acid | Energy [metabolism](https://cn.bing.com/dict/search?q=metabolism&FORM=BDVSP6&mkt=zh-cn) | ↑ |
| 23 | HMDB00630 | + | 3.58 | 112.0508 | 1.2318 | C_4_H_5_N_3_O | Cytosine | [Pyrimidine](https://cn.bing.com/dict/search?q=pyrimidine&FORM=BDVSP6&mkt=zh-cn) [metabolism](https://cn.bing.com/dict/search?q=metabolism&FORM=BDVSP6&mkt=zh-cn) | ↑ |
| 24 | HMDB00079 | + | 0.81 | 111.0539 | 1.1038 | C_5_H_8_N_2_O_2_ | Dihydrothymine | [Pyrimidine](https://cn.bing.com/dict/search?q=pyrimidine&FORM=BDVSP6&mkt=zh-cn) [metabolism](https://cn.bing.com/dict/search?q=metabolism&FORM=BDVSP6&mkt=zh-cn) | ↑ |
| 25 | HMDB02369 | + | 4.05 | 283.2015 | 3.5816 | C_20_H_28_O_2_ | 1. cis-Retinoic acid | [Retinol](https://cn.bing.com/dict/search?q=retinol&FORM=BDVSP6&mkt=zh-cn) [metabolism](https://cn.bing.com/dict/search?q=metabolism&FORM=BDVSP6&mkt=zh-cn) [Vitamin](https://cn.bing.com/dict/search?q=vitamin&FORM=BDVSP6&mkt=zh-cn) [metabolism](https://cn.bing.com/dict/search?q=metabolism&FORM=BDVSP6&mkt=zh-cn) | ↑ |
| 26 | HMDB01518 | + | 11.68 | 301.1428 | 3.05208 | C_16_H_22_O_4_ | alpha-CEHC | [Vitamin](https://cn.bing.com/dict/search?q=vitamin&FORM=BDVSP6&mkt=zh-cn) [metabolism](https://cn.bing.com/dict/search?q=metabolism&FORM=BDVSP6&mkt=zh-cn) | ↑ |
| 27 | HMDB03152 | + | 0.69 | 137.0713 | 1.2103 | C_7_H_8_N_2_O | N-Methylnicotinamid | [Vitamin](https://cn.bing.com/dict/search?q=vitamin&FORM=BDVSP6&mkt=zh-cn) [metabolism](https://cn.bing.com/dict/search?q=metabolism&FORM=BDVSP6&mkt=zh-cn) | ↑ |
| 28 | HMDB06707 | + | 10.31 | 817.5973 | 3.6611 | C_54_H_82_O_4_ | Coenzyme Q9 | [Vitamin](https://cn.bing.com/dict/search?q=vitamin&FORM=BDVSP6&mkt=zh-cn) [metabolism](https://cn.bing.com/dict/search?q=metabolism&FORM=BDVSP6&mkt=zh-cn) | ↑ |
| 29 | HMDB04194 | + | 0.90 | 153.0662 | 2.9325 | C7H8N2O2 | N1-Methyl-4-pyridone  -3-carboxamide | [Vitamin](https://cn.bing.com/dict/search?q=vitamin&FORM=BDVSP6&mkt=zh-cn) [metabolism](https://cn.bing.com/dict/search?q=metabolism&FORM=BDVSP6&mkt=zh-cn) | ↓ |
| 30 | HMDB02226 | + | 9.67 | 355.2642 | 1.0801 | C_22_H_36_O_2_ | Adrenic acid | Hormone monoamine neurotransmitter metabolism | ↓ |
| 31 | HMDB02362 | + | 11.93 | 101.0712 | 3.66737 | C_4_H_10_N_2_O_2_ | 2,4-Diaminobutyric acid | Amino acid neurotransmitter metabolism | ↑ |
| 32 | HMDB00391 | + | 8.21 | 407.2805 | 2.6227 | C_24_H_38_O_5_ | 7-ketodeoxycholic acid | Bile acid metabolism | ↓ |
| 33 | HMDB00460 | + | 12.37 | 413.2715 | 2.5729 | C_24_H_38_O_4_ | 7-Hydroxy-3-oxocholanoic acid | Bile acid metabolism | ↑ |
| 34 | HMDB01408 | + | 6.99 | 386.1248 | 1.0108 | C_13_H_24_NO_10_P | Phosphatidylserine | [Glycerol](https://cn.bing.com/dict/search?q=Glycerol&FORM=BDVSP6&mkt=zh-cn) [phosphate](https://cn.bing.com/dict/search?q=phosphate&FORM=BDVSP6&mkt=zh-cn) [metabolism](https://cn.bing.com/dict/search?q=metabolism&FORM=BDVSP6&mkt=zh-cn) | ↑ |

Note: ↑↓represents the trend of biomarkers in the model group was higher or lower than that in the blank group. HMDB (the human metabolome database) is human metabolism Group database, column to 00391, for example, on behalf of this compound in the HMDB number.
